# Immune Responses Regulated by Key Periodontal Bacteria in Germ-Free Mice

**DOI:** 10.3390/pathogens11050513

**Published:** 2022-04-26

**Authors:** Xin Shen, Yutao Yang, Jian Li, Bo Zhang, Wei Wei, Changqing Lu, Caixia Yan, Hong Wei, Yan Li

**Affiliations:** 1 State Key Laboratory of Oral Diseases, National Clinical Research Center for Oral Diseases, West China Hospital of Stomatology, Sichuan University, Chengdu 610041, China; shenxinzsl@alu.scu.edu.cn (X.S.); yangyutao@stu.scu.edu.cn (Y.Y.); hxkqww@stu.scu.edu.cn (W.W.); domiso@alu.scu.edu.cn (C.Y.); 2 Institute of Immunology, PLA, Army Medical University, Chongqing 400038, China; lijian@tmmu.edu.cn; 3 Department of Stomatology, Minda Hospital of Hubei Minzu University, Enshi 445000, China; 2004043@hbmzu.edu.cn; 4 Department of Anatomy, West China School of Basic Medical and Forensic Medicine, Sichuan University, Chengdu 610041, China; luchangqing@scu.edu.cn; 5 Central Laboratory, Clinical Medicine Scientific and Technical Innovation Park, Shanghai Tenth People’s Hospital, Tongji University, Shanghai 200435, China

**Keywords:** periodontal bacteria, innate immune, adaptive immune, germ-free

## Abstract

The immune dysregulation induced by periodontal bacteria has important roles in the development of periodontitis. However, the role of key periodontal bacteria in local and systemic immunity has not been comprehensively studied. Herein, to explore immunoregulation maps of key periodontal bacteria, a mono-colonized germ-free mice model with *P. gingivalis*, *F. nucleatum*, and *T. denticola* for two weeks was designed in this study. The alveolar bone loss was determined by micro-CT. A total of 14 types of innate and adaptive immune cells of the gingiva, spleen, and colon were detected by multi-color flow cytometry. *P. gingivalis* induced the strongest innate immune response in gingiva and mononuclear phagocytes (MNPs) changed most significantly, compared to *F. nucleatum* and *T. denticola*. Immune dysregulation of the colon was widely induced by *F. nucleatum*. *T. denticola* mainly induced immune disorder in spleen. ILC3s, Tregs, CD11B+ dendritic cells s, MNPs, macrophages, and plasmacytoid dendritic cells were the main types in response to key periodontal bacteria. However, the alveolar bone loss was not induced by key periodontal bacteria. In conclusion, the overall immunoregulation of monomicrobial stimuli to decipher the complexities of periodontitis was provided in this study. *P. gingivalis*, *F. nucleatum*, and *T. denticola* have different effects on local and systemic immunity in gingiva, colon, and spleen of germ-free mice.

## 1. Introduction

Periodontitis is a chronic inflammatory disease, which is mainly initiated by microbial dysbiosis. *Porphyromonas gingivalis* (*P. gingivalis*), *Fusobacterium nucleatum* (*F. nucleatum*), and *Treponema denticola* (*T. denticola*) are considered as the critical bacteria in microbial dysbiosis, which are highly abundant in periodontitis patients [1,2,3]. Until now, the regulation among periodontal bacteria and local inflammation/bone resorption has been summarized and widely recognized in the theory of osteoimmunology [4]. Briefly, due to a higher proportion of the periodontitis-related bacteria in subgingival plaque, the immune response is activated as well as osteogenesis-osteoclastic balance is affected [5,6]. So, how do periodontitis-related bacteria affect innate and adaptive immunity in periodontal tissue? This is a critical problem. Moreover, periodontitis is inextricably connected to systemic diseases, and for the mechanism of periodontitis microbes and distal pathogenic effects, three points were summarized in a recent study: (1) infection due to metastatic transient bacteriaemia; (2) metastatic immunological injury; and (3) metastatic toxic injury [7]. The changes of immune cell subsets in distant organs induced by periodontitis microbes from oral cavity which helps to explain the mechanisms of periodontitis affecting systemic health. In fact, the interactions between microbiota and immune system are still a hot topic in current research [8].

Innate immune response is the first line of the mucosal barrier to defend against pathogens, mainly consisting of the classical myeloid cells: monocytes (Monos), macrophages (MFs), and dendritic cells (DCs). In recent years, innate lymphoid cells (ILCs) have gradually been found to play important roles in mucosal immune responses, such as in the gastrointestinal and oral cavity [9,10]. Generally, each type of cell has its own unique functions, playing the roles of antigen presentation and activation of adaptive immune response. As the second line of host defense, the adaptive immune response mainly consists of T cells and B cells, which is bind to antigen-presenting cell receptors to induce inflammatory cytokines and antibodies. CD4+T cells (Th1, Th2, Th17, Tregs) and CD8+T cells are the most important subsets of T cells. However, very few studies have explored the comprehensive changes of innate and adaptive immune cells in the process of periodontitis [11].

Presently, studies about the roles of single periodontitis-related bacteria in modulation of local and systemic immunity are rare. Because periodontal microbiota is a complex ecosystem, it is not clear how periodontitis-related bacteria exert influence on the innate and adaptive immune systems in a side-by-side manner and how they induce immune response in the distant organs. To accurately evaluate these issues, a germ-free (GF) mice model needs to be used. The strategy of inoculating with single periodontal bacteria in GF mice followed by extensive immunophenotyping analysis could set aside the combinatorial effects of the complex microbiota, and it would help to precisely explore the interaction of mono-colonized microorganism and the host [12].

The objective is to explore how mono-colonized key periodontitis bacteria modulate the innate and adaptive immune response in local and systemic organs, with an extensive and detailed evaluation of immune cell subsets in this study. Three key periodontal bacteria strains, *F. nucleatum*, *P. gingivalis*, and *T. denticola*, were independently inoculated into the teeth of GF mice and 14 immunocytes were detected in the gingiva, colon, and spleen. This study has deciphered the complexities of immune response induced by periodontal bacteria in a side-by-side manner and offered new insights in local and systemic immune regulation for prevention and treatment of periodontitis and its associated systemic diseases.

## 2. Results

### 2.1. Bone Resorption after Inoculation with Periodontal Bacteria in Germ-Free Mice

**Figure 1:**
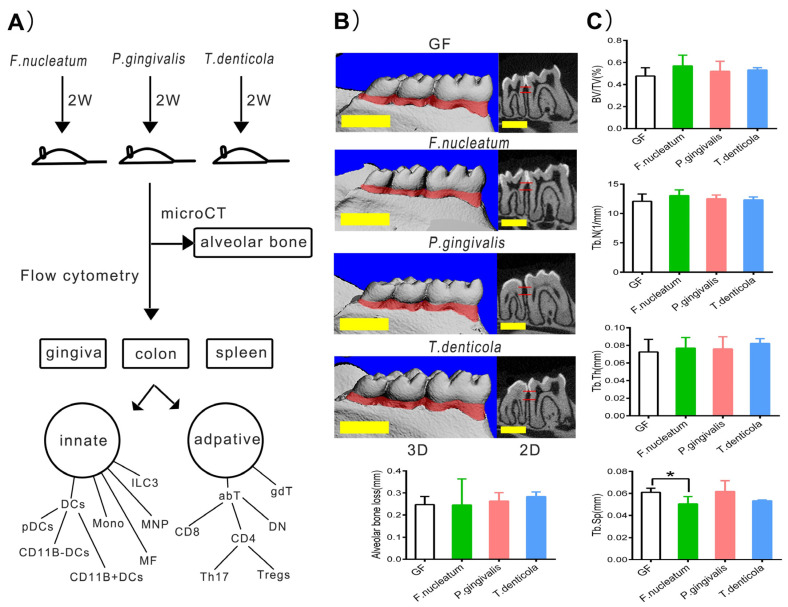
Bone resorption level after inoculation with periodontal bacteria. (**A**) Diagram of experimental design. (**B**) Reconstruction of mandibles and quantitative analysis of alveolar bone absorption. Scale bars, 1 mm. (**C**) BV/TV, Tb. Th, Tb. Sp, and Tb. N of alveolar bone. Data represents means ± SD. * *p* < 0.05. GF, germ-free mice inoculated with PBS (n = 4); *F. nucleatum*, germ-free mice inoculated with *F. nucleatum* (n = 5); *P. gingivalis*, germ-free mice inoculated with *P. gingivalis* (n = 5); *T. denticola*, germ-free mice inoculated with *T. denticola* (n = 4).

The design of the experiment is shown in Figure 1A. After inoculation with *F. nucleatum*, *P. gingivalis*, or *T. denticola* in the GF mice for two weeks, the values of the alveolar bone loss, trabecular bone volume per total volume (BV/TV), trabecular number (Tb. N), and trabecular thickness (Tb. Th) showed a little increasing trend without significant differences (*p* > 0.05), compared with those of the GF group. However, mean trabecular separation (Tb. Sp) was decreased by *F. nucleatum* (*p* < 0.05) (Figure 1B,C). Therefore, we supposed that two weeks were inadequate to induce alveolar bone resorption by periodontal bacterial infection in the GF mice model. 

### 2.2. Immunological Changes of Gingiva in Response to Periodontal Bacteria Colonization

**Figure 2:**
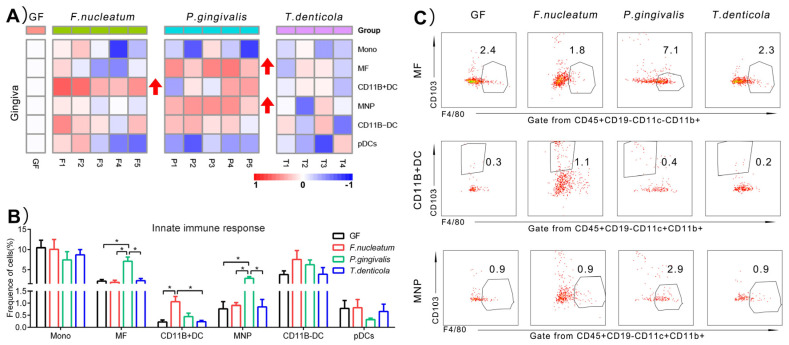
Immunological changes of gingiva in response to periodontal bacteria colonization. (**A**) Heatmap of innate immune cells changes in the gingiva. Red arrow indicated immune cells with a significant rise, compared to GF group. The mean values of the four mice in GF group represented by the one square, while the individual value of each mouse in bacteria-treated groups represented by the one square. (**B**) Histogram of the innate immune cells changes. (**C**) Representative flow cytometry dot plots showing expression of the significant difference immune cells, compared to GF group. The gate of positive immune cells was drawn by frames. The frequency of gated immune cells in each group was showed by the mean values on the dot plots. A higher density cell populations is represented by the “blue color” and those with a lower density are represented by the “red color”. The medium density of cell populations is represented by the “green or yellow color”. Every dot represents a single cell. Data represents means ± SD. * *p* < 0.05.

To provide a baseline of gingiva involved in periodontal bacteria colonization, a small-scale test characterizing the major shifts in the innate immune cells was performed in Figure 2A. Monos, MFs, and DCs remained a smaller population in gingiva (<10%) (Figure 2B). Compared to GF group, the percentage of MFs (7.1 ± 2.4%, *p* < 0.05) and mononuclear phagocytes (MNPs) (2.9 ± 0.8%, *p* < 0.05) was significantly increased, reflecting a three-fold increase after inoculation with *P. gingivalis* in GF mice. Approximately 1.1 ± 0.5% (*p* < 0.05) CD11B+ dendritic cells (CD11B+DCs) had been identified in gingiva when encountering *F. nucleatum* colonization, with a three-fold increase (Figure 2C). However, the frequencies of Monos, CD11B- dendritic cells (CD11B-DCs), and plasmacytoid dendritic cells (pDCs) did not appear to significantly change due to periodontal bacteria (Figure 2B). 

### 2.3. Immunological Changes of Colon in Response to Periodontal Bacteria Colonization

**Figure 3:**
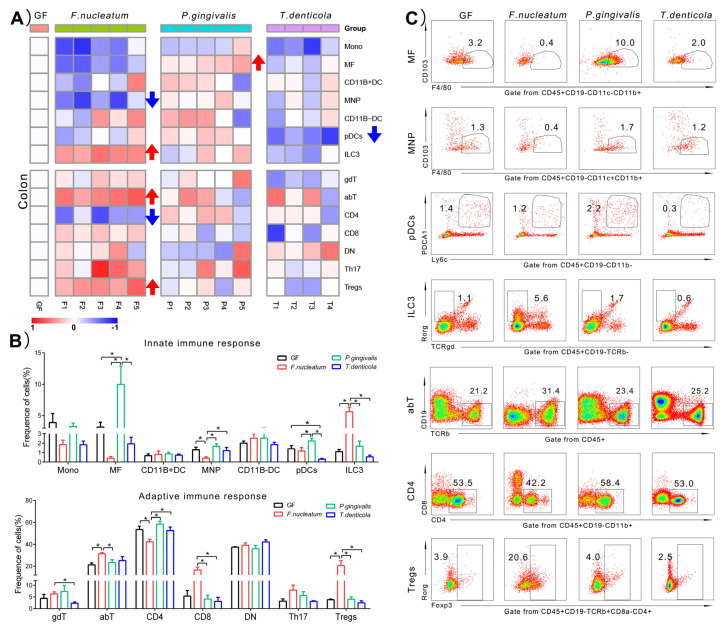
Immunological changes of colon in response to periodontitis bacteria colonization. (**A**) Heatmap of innate immune cells changes in colon. Blue arrow indicated immune cells with a significant rising, compared to GF group. (**B**) Histogram of the innate immune cells changes. (**C**) Representative flow cytometry dot plots showing expression of the significant difference immune cells, compared to GF group. The gate of positive immune cells was drawn by frames. The frequency of gated immune cells in each group was showed by the means on the dot plots. A higher density cell populations is represented by the “blue color” and those with a lower density are represented by the “red color”. The medium density of cell populations is represented by the “green or yellow color”. Data represents means ± SD. * *p* < 0.05.

We also investigated whether orally colonized with periodontal pathogens would provoke the change of immune response in the guts. A total of 14 types of immune cells of colon were illustrated in Figure 3. Generally speaking, a larger type of immune cells were in response to *F. nucleatum* in the colon, especially in terms of adaptive immune cells, compared with the other two bacteria (Figure 3A). Among the innate immune cells, the proportion of MNPs significantly decreased from 1.3 ± 0.6% to 0.4 ± 0.3% (*p* < 0.05), whereas the proportion of group 3 innate lymphoid cells (ILC3s) significantly increased from 1.1 ± 0.5% to 5.6 ± 1.5% (*p* < 0.05), compared to the GF group. For adaptive immune cells, the total proportion of αβT cells (abT) cells increased from 21.2 ± 3.6% to 31.4 ± 2.5% (*p* < 0.05) in the colon. Further analysis of subsets of abT cells demonstrated that CD4+T cells were downregulated by 20% from 53.5 ± 6.1% to 42.2 ± 5.5% (*p* < 0.05), compared to the GF group. However, the percentage of CD8+T cells increased with no significant difference (*p* = 0.08), and a five-fold upregulation was observed in the colonic Tregs, from 3.9 ± 0.5% to 20.6 ± 10.1% (*p* < 0.05). 

However, a lot of colonic immune cells were not responsive to *P. gingivalis* and *T. denticola*. The frequency of MFs was increased nearly three times by *P. gingivalis*, from 3.2 ± 1.5% to 10.0 ± 6.4% (*p* < 0.05), compared with the GF group. The most innate immune cells of colon had a downregulated trend by *T. denticola*, especially, the proportion of pDCs significantly decreased, from 1.4 ± 0.7% to 0.3 ± 0.2% (*p* < 0.05). 

### 2.4. Immunological Changes of Spleen in Response to Periodontal Bacteria Colonization

**Figure 4:**
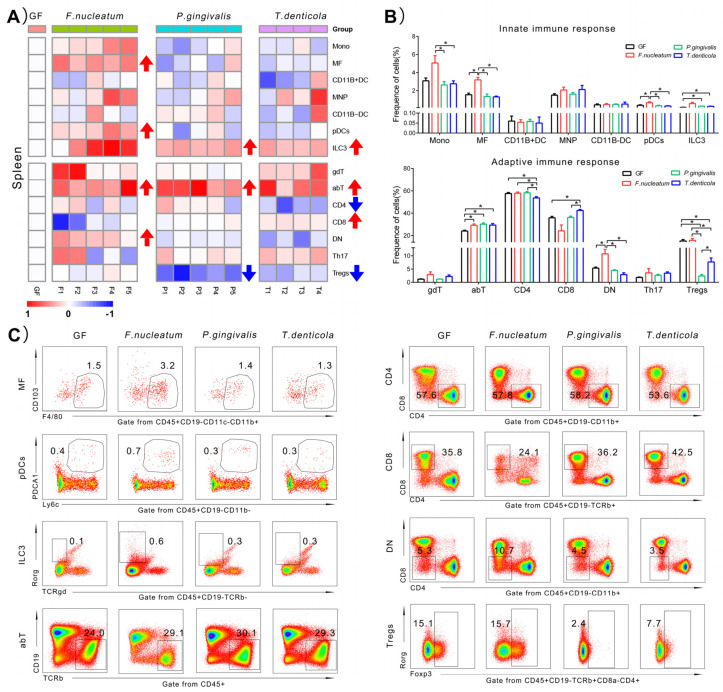
Immunological changes of spleen in response to periodontitis bacteria colonization. (**A**) Heatmap of innate immune cells changes in spleen. (**B**) Histogram of the innate and adaptive immune cells changes. (**C**) Representative flow cytometry dot plots showing expression of the significant difference immune cells, compared to GF group. The gate of positive immune cells was drawn by frames. The frequency of gated immune cells in each group was showed by the means on the dot plots. A higher density cell populations is represented by the “blue color” and those with a lower density are represented by the “red color”. The medium density of cell populations is represented by the “green or yellow color”. Data represents means ± SD. * *p* < 0.05.

To further explore the systemic immune landscape induced by periodontal bacteria, the changes of immune cells of spleen were analyzed. As shown in Figure 4A, a larger numbers of immune cell types changed in the spleen than in the colon, induced by periodontal bacteria. The proportions of the ILC3s and abT cells were widely rising while Tregs were downregulated by periodontal bacteria. The immune cells response to *T. denticola* was much larger in the spleen than gingiva and colon.

For mono-colonization with *F. nucleatum*, four types of immune cells increased, including the proportions of MFs (from 1.5 ± 0.4% to 3.2 ± 0.6%, *p* < 0.05), pDCs (from 0.4 ± 0.1% to 0.7 ± 0.2%, *p* < 0.05), abT cells (from 24.0 ± 1.5% to 29.1 ± 2.7%, *p* < 0.05), and CD4-CD8- T cells (DN)(from 5.3 ± 0.8% to 10.7 ± 3.7%, *p* < 0.05), compared with those of the GF group. 

After inoculation with *P. gingivalis*, a slight increase was found in proportion of ILC3s (from 0.1 ± 0.03% to 0.3 ± 0.03%, *p* < 0.05) and abT cells (from 24.0 ± 1.5% to 30.1 ± 2.6%, *p* < 0.05), while a near seven-fold decrease in proportion of Tregs (from 15.1 ± 2.5% to 2.4 ± 1.2%, *p* < 0.05), compared with those of the GF group.

Adaptive immune cells were more responsive than innate ones of the spleen after inoculation with *T. denticola*, at least in terms of the frequencies. There was an increase in the proportion of ILC3s (from 0.1 ± 0.03% to 0.3 ± 0.03%, *p* < 0.05) induced by *T. denticola*. As the dominant T cell subsets, abT cells represented about 30% and they were upregulated from 24.0 ± 1.5% to 29.3 ± 2.8% (*p* < 0.05) in *T. denticola* group. Among the total numbers of abT cells, the proportion of CD8+ T cells increased 19%, from 35.8 ± 2.4% to 42.5 ± 1.7% (*p* < 0.05). However, one other important subset of abT cells, the proportion of CD4+ T cellswas decreased slightly, from 57.6 ± 1.7% to 53.6 ± 2.2% (*p* < 0.05). Furthermore, of total CD4+ T cells, Tregs were decreased by two times, from 15.1 ± 2.5% to 7.7 ± 3.0% (*p* < 0.05), compared with those of the GF group.

### 2.5. Summary of the Significantly Changing Immune Cell Types

Taking together, these data from all the heatmaps in Figure 2, Figure 3 and Figure 4 indicated that: (1) in the gingiva, the proportions of MFs, MNPs, and CD11B+DCs were significantly rising; (2) in the colon, the proportions of MFs, MNPs, pDCs, ILC3s, abT cells, CD4+T cells, and Tregs were significantly changed; (3) in the spleen, similarly, the proportions of MFs, pDCs, ILC3s, abT cells, CD4+T cells, CD8+T cells, DN cells, and Tregs showed significant changes after mono-infection of three key periodontal bacteria, compared with those of the GF group. The above results can be summarized in Table 1. Furthermore, due to the change of the frequencies over three times, we speculated that ILC3s, Tregs, CD11B+DCs, MNPs, MFs, and pDCs induced by three key periodontal bacteria were probably more related to the immune responses in the three organs than other immunocytes. 

## 3. Discussion

Periodontitis is a chronic inflammatory disease that results in the destruction of periodontal tissues and tooth loss. The continuous inflammation and bone resorption are related to the host’s immune disorders, which can be ascribed to microbial dysbiosis of the oral environment. Our previous research revealed that *Fusobacteria*, *Porphyromonas*, and *Treponema* were most closely associated with the severe periodontitis patients by 16s rRNA gene sequencing analysis of subgingival plaque [13]. Thus, *F. nucleatum*, *P. gingivalis*, and *T. denticola* were selected as the key periodontal bacteria to mono-colonize GF mice in this study. A total of 14 immune cells in the gingiva, spleen, and colon after oral infection of key periodontitis bacteria were comprehensively detected to broadly explore their influence on local and systemic immune response. We found that the alveolar bone resorption was not induced after bacterial infection for two weeks. However, the local and systemic immune responses were triggered as early as two weeks. We speculated that the early intervention of immune dysfunction could probably reverse the subsequent periodontal destruction in periodontitis patients. Some interesting results were discussed as follows. 

### 3.1. P. gingivalis Induced a Higher Innate Immune Response in the Gingiva, Compared to F. nucleatum and T. denticola

The actual role of *P. gingivalis* has been redefined as the keystone pathogen in periodontitis-associated bacteria recently. *P. gingivalis* has an important role in the conversion from homeostasis to dysbiosis of the microbial community [14]. It utilizes a variety of virulence factors to cause deregulation of the innate immune and inflammatory responses in the periodontal tissues [15]. Lipopolysaccharide of *P. gingivalis* is a stimulator of proinflammatory responses, which could recruit macrophages into the periodontal tissues [16]. Otherwise, if the depletion of macrophages happened, the alveolar bone loss of mice would be inhibited [17]. We also demonstrated that the frequency of macrophages increased by *P. gingivalis* in GF mice model. Moreover, it is crucial that the immune response is activated by mononuclear phagocytes (DCs, MFs, MNPs) against the pathogens [18]. The role of DCs and macrophages in periodontitis has been studied [19], while whether MNPs could be in response to periodontal bacteria is currently unknown. Our results showed a significant increase in proportions of macrophages and MNPs of gingiva by *P. gingivalis*, whereas the other two bacteria exerted no immunological effect on gingiva. It was considered *P. gingivalis* as probably the most critical bacteria in the gingiva in our study. The functions of macrophages and MNPs in *P. gingivalis*-related periodontitis could be highlighted in further study.

### 3.2. The Local Immunity of the Colon Induced by F. nucleatum Was More Responsive Than P. gingivalis and T. denticola

In this study, a significant alteration of immune cells in the colon was caused by *F. nucleatum*, compared to the other two bacteria. Previous studies stated that a higher detection rate of *F. nucleatum* was found in colorectal cancer tissues [20]. Furthermore, in the saliva sample applied to GF mice, *F. nucleatum* were detected in the content of colon [21]. A much stronger immune response was activated might be consistent with the specialized ability of *F. nucleatum* to colonize the colon. Under the colonic cancer state, *F. nucleatum* could promote the recruitment of myeloid immune cells (CD11B+ cells) and inhibit the CD4+T cells [20]. Our study suggested that *F. nucleatum* derived from the oral cavity had a significant effect on the proportions of both innate immune and adaptive immune cells, with decreased proportions of MNPs and CD4+T cells and increased proportions of ILC3s, abT cells, and Tregs. The types of innate immune cells infiltration in the colon induced by *F. nucleatum* are probably different between the condition of periodontitis and colon cancers. Therefore, more evidence is needed to explain the immune regulation of *F. nucleatum* in the colon, which it might provide a bridge between periodontitis and colon cancers. 

### 3.3. One of the Main Effects Induced by T. denticola Was on the Systemic Immune Response

From the heatmaps, we found that *T. denticola* mainly deregulated the proportions of immune cells in the spleen and had fewer effects on local immune response, compared with *P. gingivalis* and *F. nucleatum*. There are a lot of virulence factors of *T. denticola* in relation to the inhibition or activation of immune cells (major outer sheath protein) [22]. As one secretary virulence factors of *T. denticola*, the outer membrane vesicles might penetrate blood vessels [23]. Furthermore, the DNA of *T. denticola* was detected in the spleen of mono-infected SCID mice [24]. Taken together, it might be in relation to the much stronger systemic immune effect induced by *T. denticola* in GF mice. It was also found that *T. denticola* inhibited the proliferation of human peripheral blood lymphocytes in response to both mitogen and antigens *in vitro* [25]. In fact, Jones et al. showed that *T. denticola* could modify phosphoinositide signaling to repress neutrophil signaling and overall dampen the immune response, which might be the mechanisms of the impaired neutrophil functions in patients with periodontitis [26]. Similarly, our study also found that in GF mice, *T. denticola* mainly downregulated pDCs in colon.

### 3.4. The Key Immune Cells in Response to Mono-Colonization of Key Periodontal Bacteria

Our study revealed that six types of key immune cells in response to mono-colonization of key periodontal bacteria, namely ILC3s, Tregs, CD11B+DCs, MNPs, MFs, and pDCs. Among those cells, the proportions of ILC3s and Tregs changed more than five times in local or systemic organs, which would be the future research directions for immunoregulation between periodontal bacteria and the host. The role of ILC3s in mucosal immune homeostasis has been gradually realized in recent years. ILC3s are regulated by cytokines, which are produced by mononuclear phagocytes or triggered by intestinal microbiota [27]. ILC3s are also the predominant subset in the periodontal tissue of periodontitis, and they were activated by producing more IL-17A and IFN-γ in comparison with the healthy ones [28]. In our study, ILC3s were increased by *F. nucleatum*, thus we speculated that ILC3s might participate in the progression of periodontitis. Tregs play important roles in immunodepression and immune homeostasis [29]. Increased numbers of Tregs protected the host from microbial infection [30]. Th17 and Tregs were considered two important subtypes of CD4+T cells. The ratio of Th17/Tregs manifested the progression of inflammation. *P. gingivalis* could suppress adaptive immunity by modulation of the Th17/Treg imbalance [31]. Our study suggested that in GF mice, Tregs were mainly suppressed in the spleen by periodontal bacteria. Thus, Tregs are probably critical immune cells after infection of *P. gingivalis* or *T. denticola* from the oral cavity. 

### 3.5. Direction for Future Research

Firstly, the key immune cells in response to mono-colonization of periodontal bacteria could be markers to evaluate the severity of periodontitis, as well as to predict the potential role of some systemic diseases. Targeting the key immune cells may be a therapeutic direction in the future. Secondly, the function of key types of immune cells in local and systemic organs should be further studied to explain the relationship between periodontal bacteria and systemic diseases. However, the present data in our study are limited because only three species of periodontal bacteria were used. Thus, in the future, in order to decipher the complexities of immune response induced by periodontal bacteria in a side-by-side manner, much more species abundant in periodontitis should be included to colonize the GF mice. We screened 18 strains of 15 species bacteria (preserved by our lab) from 8 genera for subsequent studies, which were: *Bacteroidota* (*Porphyromonas*, *Tanella*, *Prevotella*, *Bacteroides*, *Capnocytophaga*), *Fusobacteria* (*Fusobacterium*, *Leptothrix*), and *Spirochaetes* (*Treponema*). *Actinomycete* served as an alternative. We would like to establish a robust, “sensitized” readout system that permits screening for periodontitis-derived immunomodulatory bacteria. Moreover, with the limitation by the complexity of the composition of oral microbiota, further studies involving orally colonized with saliva samples from periodontitis patients are necessary to define the role of microbiota-immune interactions in periodontitis-associated extra-oral diseases.

## 4. Materials and Methods

### 4.1. Bacteria and Mice

*P. gingivalis* (W83), *F. nucleatum* (ATCC25586), and *T. denticola* (ATCC35405) were obtained from the State Key Laboratory of Oral Disease, Sichuan University. *P. gingivalis* and *F. nucleatum* were cultured overnight in Brain Heart Infusion medium supplemented with hemin and vitamin K, and *T. denticola* was grown in new oral spirochete medium as described previously [32] under strict anaerobic conditions (80% N_2_, 10% H_2_, 10% CO_2_) at 37 °C. 

A total of 19 GF BALB/c mice (5 weeks old, females) were provided by the Laboratory Animal Center of Army Medical University, Chongqing. These GF mice models were established by rearing and breeding the pups in sterile isolators. The food and water were autoclaved to maintain sterility. Those mice were randomly allocated into four groups: GF (control germ free mice, n = 4); *F. nucleatum* (inoculated with *F. nucleatum*, n = 5); *P. gingivalis* (inoculated with *P. gingivalis*, n = 5), and *T. denticola* (inoculated with *T. denticola*, n = 5), and housed under a 12-h light-dark cycle with free access to food and water at room temperature. More details to keep and check the mice sterile were described in Appendix A.

### 4.2. Animal Model

The Ethics Committee of the West China Hospital of Stomatology, Sichuan University (WCHSIRB-D-2017-069) approved this study. Mice were orally inoculated with *F. nucleatum*, *P. gingivalis*, or *T. denticola*. Bacterial inoculation was conducted twice during the two weeks, at the 1st and 2nd week. For each mouse, 10^10^ GFU/mL mixed in 200 μL PBS of each single bacterial strain were applied on all the teeth. One mouse died after inoculation *T. denticola* for one week and was excluded from the experiment. The remaining 18 mice were euthanized after two weeks infection. The feces of all mice were collected and cultured in media, which were identified by Gram staining and microscopy each week to ensure conditions without contamination. Before all mice euthanized, live *F. nucleatum* and *P. gingivalis* were successfully detected in the two groups. However, live *T. denticola* was not detected in fecal samples. 

### 4.3. Micro-CT Scanning of Alveolar Bone Resorption

The left mandibles from all the mice were removed and fixed in 4% paraformaldehyde for 24 h. The samples were evaluated using a micro-CT scanner (Viva CT40, Scanco Medical, Wangen-Brüttisellen, Switzerland). Scans were performed at a resolution of 10 μm. The images were analyzed by 3D reconstruction. The volume of interest for calculation was defined as the alveolar bone from the cemento-enamel junction to the apex of first molar teeth for a series of 30 slices. Five parameters were calculated: alveolar bone loss, BV/TV, Tb.N, Tb.Sp, and Tb.Th. 

### 4.4. Immune Cell Preparation and Multi-Color Flow Cytometry Analysis

Gingival tissues were collected from the first molars to the third molars of maxilla bone after the mice being perfused with PBS. Because of the thick keratin layer and nasal associated lymphoid tissue, these gingival tissues from the hard palate were not included. Single cell suspensions of the gingiva, colon, and spleen were prepared as previously described [12,33]. 

Before cell staining, the suspensions were washed and filtered through a strainer (70 μm). Anti-mouse CD16/32 antibody was used for blocking the Fc domain before staining for the surface or intracellular markers. The protocol and gating strategy of 14 types of immune cells was described in Figure S1. Antibodies were divided into two groups, the first group represented by the CD45, CD19, CD11c, CD11b, F4/80, CD103, Ly6c, and PDCA1 for evaluation of innate immune cells; the second group represented by the CD45, CD19, TCRß, TCRγδ, CD4, CD8, Foxp3, and Rorγ for evaluation of adaptive immune cells. The labeling fluorochrome and numbers of the mAbs were listed in Table S1. Due to the limited cells, the suspensions of gingiva were incubated with only the first group of antibodies. The suspensions of colon and spleen samples were incubated with both groups. Cells were incubated with the antibodies following the manufacturer’s protocol. All the samples were examined by a flow cytometer (BD Celesta, Becton, Dickinson and Company, New Jersey, USA). Analysis was performed using the Kaluza software. The proportions of immune cell subsets were calculated. The proportions of the following 14 immune cells were analyzed: Monos, MFs, MNPs, CD11B+DCs, CD11B-DCs, pDCs, ILC3s, gdT, abT, CD4-CD8- T cells, DN, CD8+ T cells, CD4+ T cells, Th17, and Tregs. The definition and gating of immune cells are shown in Table S2, which is described in the previous study [12].

### 4.5. Drawing of Heatmap

The results of the multi-color flow cytometry analysis were processed as following steps: firstly, the fold change cell values compared to GF log2 value were calculated of all immune cell subsets in individual mouse; then, these values of each subset were normalized to a scope of [−1,1], using the formula: relative change = X/(X_max_ − X_min_). Finally, a heatmap was created using the R project.

### 4.6. Statistical Analysis

SPSS (version 24.0; IBM Corporation, Armonk, NY, USA) was used for statistical analyses. Data are shown as means ± SD. ANOVA was used to compare the differences between four groups. *p* value < 0.05 was considered significant.

## 5. Conclusions

In this study, three key periodontal bacteria exerted significantly different effects on the innate and adaptive immunity in local and systemic organs. This highlights the importance of bacterial specificity being associated with immunophenotypes. The periodontal bacterial immune subversion would tip the balance from homeostasis to disease in oral or extra-oral sites.

## Figures and Tables

**Table 1:** The significantly changing immunocytes in response to key periodontal bacteria.

Organs	*F. nucleatum*	*P. gingivalis*	*T. denticola*
Gingiva	CD11B+DC↑	MNPs↑, MFs↑	/
Colon	ILC3s↑, MNPs↓ Tregs↑, abT↑ CD4+T↓	MFs↑	pDCs↓
Spleen	MFs↑, pDCs↑ DN↑, abT↑	ILC3s↑ Tregs↓, abT↑	ILC3s↑ abT↑, CD8+T↓ CD4+T↓, Tregs↓

Notes: Compared to control group, the immune cells that were < 2 times in the bacteria inoculation group was marked in green, ≥2 times in blue, ≥3 times in black, ≥5 times in red. ↑ means increasing, ↓ means decreasing.

## Data Availability

The data generated/analyzed for this study can be made available from the corresponding author upon reasonable request.

## Supplementary Materials

The following supporting information can be downloaded at: https://www.mdpi.com/article/10.3390/pathogens11050513/s1, Figure S1: The gating strategies; Table S1: The list of antibodies used in the multicolor flow cytometry. Table S2: Protocol of gating in flow cytometry.

## Appendix A

All equipment, food, bedding, and water were sterilized by autoclaving. Material entering the isolator were sprayed with 2% per-acetic acid. All operations were performed under sterile conditions throughout life cycle including death in GF model. Fecal and surface swab samples from isolators were cultured once a week by using both aerobic and anaerobic incubation conditions. No bacteria could be cutured from the noninfected control. No contamination microorganisms were found during culture of the isolators at any time throughout the study. The sterility of GF animals was examined by aerobic and anaerobic culture of oral swabs and fecal pellets on non-selective media and by PCR using universal 16S primers.
