# Supplementary material for: Immune Responses Regulated by Key Periodontal Bacteria in Germ-Free Mice
Source: Pathogens. 2022 Apr 26;11(5):513. doi: 10.3390/pathogens11050513 (PMC9146732; doi:10.3390/pathogens11050513)
Supplement: Supplementary file 1 [file pathogens-11-00513-s001.zip › pathogens-1676556-supplementary.pdf]

Supplementary Material

Immune Responses Regulated by Key Periodontal Bacteria in Germ-Free Mice

Xin Shen [1,†], Yutao Yang [1,†], Jian Li [3], Bo Zhang [4], Wei Wei [1], Changqing Lu [5], Caixia Yan [1], Hong Wei [2,*] and Yan Li [1,*]

1 State Key Laboratory of Oral Diseases, Department, National Clinical Research Center for Oral Diseases, West China Hospital of Stomatology, Sichuan University, Chengdu 610041, China; shenxinzsl@alu.scu.edu.cn (X.S.); yangyutao@stu.scu.edu.cn (Y.Y.); hxkqww@stu.scu.edu.cn (W.W.); domiso@alu.scu.edu.cn (C.Y.); feifeiliyan@scu.edu.cn (Y.L.)

2 Central Laboratory, Department, Clinical Medicine Scientific and Technical Innovation Park, Shanghai Tenth People's Hospital, Tongji University, Shanghai 200435, China; weihong63@mail.sysu.edu.cn (H.W.)

3 Department, Institute of Immunology, PLA, Army Medical University, Chongqing 400038, China; lijian@tmmu.edu.cn

4 Department of Stomatology, Minda Hospital of Hubei Minzu University, Enshi 445000, China; 2004043@hbmzu.edu.cn

5 Department of Anatomy, West China School of Basic Medical and Forensic Medicine, Sichuan University, Chengdu 610041, China; luchangqing@scu.edu.cn

[*] Correspondence: weihong63@mail.sysu.edu.cn (H.W.); feifeiliyan@scu.edu.cn (Y.L.).

[†]These authors contributed equally to this work.

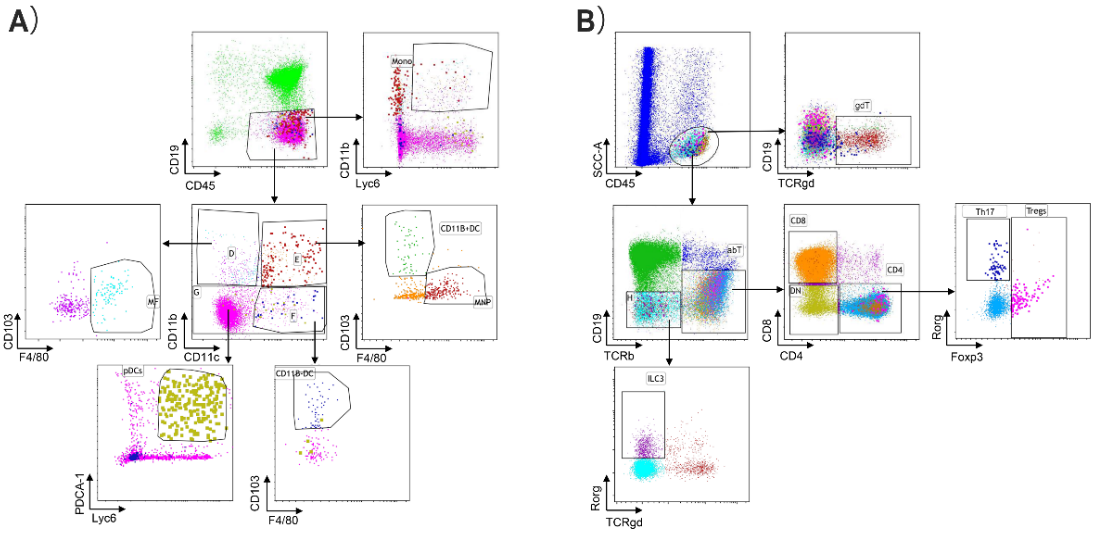

Figure S1. The gating strategies.(A) innate immunity. (B) Adaptive immunity.

Table S1. The list of antibodies used in the multicolor flow cytometry

Antidodies	Source	Identifier
Anti-mouse CD45 Brilliant Violet 605	Biolegend	Cat#103140
Anti-mouse CD11c PE Cy7	Biolegend	Cat#117318
Anti-mouse/human CD11b Percp Cy5.5	Biolegend	Cat#101228
Anti-mouse Ly6c FITC	Biolegend	Cat#128006
Anti-mouse F4/80 Alexa 700	Biolegend	Cat#123130
Anti-mouseCD137(PDCA-1) Alexa Fluor 647	Biolegend	Cat#127106
Anti-mouse CD103 PE	Biolegend	Cat#121406
Anti-mouse CD19 APC Cy7	Biolegend	Cat#115530
Anti-mouse CD45 Pacific blue	Biolegend	Cat#103126
Anti-mouse CD4 FITC	Biolegend	Cat#100406
Anti-mouse CD8a Alexa 700	Biolegend	Cat#100730
Anti-mouseTCRβchain PE Cy7	Biolegend	Cat#109222
Anti-mouseTCRgd Percp Cy5.5	Biolegend	Cat#118118
Anti-mouse Foxp3 APC	Affymetrix/eBioscience	Cat#17-5773-82
Anti-mouse ROR gamma(t) PE	Affymetrix/eBioscience	Cat#12-6988-80

Table S2. Protocol of gating in flow cytometry.

Cell name in figures	Full cell name	Definition	Reported as % of
Mono	Monocytes	Ly6c+CD11b+CD45+cd19-	CD45+CD19-
MF	CD11b+CD11c-F4/80+ macrophages	F4/80+CD103-CD11b+CD11c-CD45+CD19-	CD45+CD19-
MNP	CD11b+CD11c+F4/80+ mononuclear phagocytes	F4/80+CD103-CD11b+CD11c+ CD45+CD19-	CD45+CD19-
CD11B+DC	CD103+CD11b+ dendritic cells	F4/80CD103+CD11b+CD11c+ CD45+CD19-	CD45+CD19-
CD11B-DC	CD103+CD11b- dendritic cells	F4/80-CD103+CD11b-CD11c+ CD45+CD19-	CD45+CD19-
pDCs	plasmacytoid dendritic cells	PDCA1+Lyc6+ CD11b-CD45+CD19-	CD11b-CD19-CD45+

ILC3	Innate lymphocytes type 3	Rorg+CD45+TCRb-CD19-TCRgd-	CD45+
gdT	TCRgd T cells	TCRgd+CD45+CD19-	CD45+
abT	TCRab T cells	TCRb+CD45+CD19-	CD45+
DN	CD4-CD8- double negative T cells	CD4-CD8a-TCRb+CD19-CD45+	TCRb+CD45+CD19-
CD8	CD8+ T cells	CD8a+CD4-TCRb+CD19-CD45+	TCRb+CD45+CD19-
CD4	CD4+ T cells	CD4+CD8a-TCRb+CD19-CD45+	TCRb+CD45+CD19-
Th17	Rorg+ conventional T cells	Rorg+Foxp3-CD4+CD8a-TCRb+CD19-CD45+	CD4+CD8a-TCRb+CD19-CD45+
Tregs	Foxp3+ regulatory T cells	Foxp3+CD4+CD8a-TCRb+CD19-CD45+	CD4+CD8a-TCRb+CD19-CD45+
